# New Developments, Challenges and Open Questions in Diagnosis and Treatment of Gestational Diabetes Mellitus

**DOI:** 10.3390/jcm11237197

**Published:** 2022-12-03

**Authors:** Tina Linder, Iris Dressler-Steinbach, Andrea Tura, Christian Göbl

**Affiliations:** 1 Department of Obstetrics and Gynaecology, Medical University of Vienna, 1090 Vienna, Austria; 2 Clinic of Obstetrics, Charité-Universitätsmedizin Berlin, 13353 Berlin, Germany; 3 CNR Institute of Neuroscience, 35127 Padova, Italy

## 1. Introduction

The prevalence of gestational diabetes mellitus (GDM) is increasing alongside a rising maternal age at conception, an increasing number of people making unhealthy lifestyle choices and, especially, an increasing pregestational body weight [1]. Thereby, the global prevalence of GDM (defined as diabetes that first occurs in pregnancy and is not clearly type 1 or type 2 diabetes) is around 14%, with a strong variation across ethnic populations and which depends on the diagnosis criteria used in different countries [2]. More than half a century ago, Pedersen postulated that persistent maternal hyperglycaemia is associated with structural changes due to a greater uptake of glucose in the growing fetus [3]. Since then, maternal hyperglycaemia associated with GDM has still been widely accepted as a major risk factor for the development of macrosomia and adverse maternal and perinatal outcomes. However, despite major advances in basic and clinical science over the past decades, there are still a number of controversies and open questions, which we will briefly address in the following sections of this commentary.

## 2. GDM Testing and Risk Stratification in Early Pregnancy

Most healthcare organisations recommend GDM testing between 24 and 28 weeks of gestation via either a 75 g Oral Glucose Tolerance Test (OGTT; one-step procedure), or a non-fasting 50 g Glucose Challenge Test (GCT) followed by a 100 g OGTT (two-step procedure) [4,5]. One recent randomised controlled trial found no differences regarding perinatal and maternal complications between both testing strategies, although the prevalence of GDM was markedly higher in women using the one-step approach [6]. Although the advantages and disadvantages of both strategies require further investigation and discussion [7], there is even less consensus on whether GDM should be tested or diagnosed before 24 weeks of gestation [8]. The validity of an OGTT at the beginning of pregnancy is controversially and a matter of ongoing research [9]. Alternatively, anamnestic risk factors, such as maternal age, overweight or obesity status, ethnicity and, especially, a history of GDM in a previous pregnancy can be used to distinguish women already at the early gestation stage who have a low or high risk for the later development of hyperglycaemia [10]. These risk factors can be easily assessed and their predictive performance can be further improved via clinical prediction models, using the combined information of several anamnestic and laboratory parameters (such as fasting glucose, triglycerides or HbA1c). In a recent validation study, Kotzaeridi et al. assessed a total of 15 published risk assessment tools used during early pregnancy in order to predict the later development of GDM. The most published prediction models showed an acceptable accuracy in terms of discrimination (i.e., the ability of a model to separate pregnant women with the disease from those without the disease), although the model calibration (i.e., the agreement between predicted vs. observed probability of having the disease) was sometimes poor, depending on the different diagnostic guidelines used in the original studies [11]. A further analysis within Kotzaeridi’s study underlined the relevance of biochemical variables in order to improve prediction [11], suggesting that future research on GDM-specific biomarkers is required to provide an accurate risk stratification at the start of pregnancy [8].

## 3. Phenotypic Heterogeneity of GDM

Once diagnosed, GDM is often treated as a homogenous disease, although recent evidence suggests that different underlying metabolic factors contribute to the heterogeneous clinical picture of GDM [12,13]. In a recent study, we classified GDM subtypes according to the occurrence of fasting and post-load hyperglycaemia during the diagnostic OGTT at mid-gestation [14]; thereby, women with isolated fasting hyperglycaemia as well as those with combined hyperglycaemia (i.e., elevated fasting and post-load glucose) showed distinct metabolic alterations including impaired insulin action and were more likely to receive insulin treatment as compared to those patients with isolated post-load hyperglycaemia. However, we failed to identify differences in obstetric outcomes such as the rate of large-for-gestational-age (LGA) offspring. In another retrospective study, Linder et al. classified mothers with GDM according to their pregestational BMI as normal weight, overweight and obese patients, and observed that elevated BMI was closely associated not only with the requirement of pharmacotherapy (insulin and/or metformin), but also with an increased risk of LGA newborns and caesarean section delivery [15]. Most interestingly, the incidence of LGA was highest in obese mothers that needed glucose-lowering medications. This may be due to either poor glycaemic control despite pharmacotherapy or, alternatively, to pathophysiological alterations, which can trigger both fetal overgrowth and the treatment failure of lifestyle modifications. Taken together, these results suggest that future research on the phenotypic heterogeneity of GDM is necessary to elaborate personalised treatment approaches. These should incorporate information about fasting and post-load hyperglycaemia, as well as maternal overweight or obesity status.

## 4. The Possible Advantage of Continuous Glucose Monitoring

Continuous glucose monitoring (CGM) informs users about their current glucose levels and, hence, allows for timely adaptations before upper and/or lower thresholds are reached. This immediate feedback can improve glycaemic control without increasing the risk of hypoglycaemia in non-pregnant patients with type 1 or type 2 diabetes [16]. However, the effectiveness of CGM during pregnancy has been less well investigated. One major randomised controlled trial showed that pregnant women with type 1 diabetes randomised to real-time CGM spent more time in the target range (i.e., 63 to 140 mg/dL, or 3.5 to 7.8 mmol/L) and less time with hyperglycaemia as compared to the control group receiving routine care (i.e., capillary self-monitored blood glucose via finger prick test), whereas no differences were observed for the frequency or time spent with hypoglycaemia. Most importantly, neonatal outcomes were significantly improved in the CGM group, with a lower incidence of LGA offspring, fewer neonatal intensive care admissions and a lower incidence of neonatal hypoglycaemia [17]. Furthermore, a non-randomised study indicated improved glycaemic control and reduced glycaemic variability as well as improved pregnancy outcomes associated with the intermittent use of CGM in patients with GDM [18]. However, the use of an older version of a CGM device (i.e., a “retrospective” CGM, where glucose levels were not directly visible for the patients) may be a major limitation of this observational study. Another randomised controlled study conducted in different European centres and that is adequately powered for perinatal outcomes (especially differences in LGA incidence) is actually ongoing and will provide further insights into the possible advantages and limitations of CGM use in pregnancies with GDM [19].

## 5. Future Perspectives—Is It Time to Change the Glucocentric View?

There is emerging evidence that an increased maternal BMI is related to fetal overgrowth even in mothers with normal glucose tolerance [20]. This association may be triggered by maternal insulin resistance and compensatory hyperinsulinemia, which possibly affects the feto-placental unit and promotes fetal overgrowth already in the early pregnancy stage [21]. This hyperinsulinemic environment in insulin-resistant, obese mothers may also be linked to other perinatal complications (such as preeclampsia), consequently leading to poor obstetric outcomes as observed for overweight or obese mothers independently of maternal glycaemia [22]. Therefore, a more detailed investigation of pathophysiological alterations associated with an exceeding maternal BMI will be necessary. Since lifestyle interventions [23], as well as pharmacotherapy with metformin [24,25], failed to improve pregnancy outcomes in obese pregnancies without diabetes, future research should focus on strategies optimising preconception care and pregestational weight in obese women before conception.
